# Cortical symptoms described in emergency calls for patients with suspected large vessel occlusion: a descriptive analysis of 157 emergency calls

**DOI:** 10.1186/s12873-022-00706-5

**Published:** 2022-08-13

**Authors:** Pauli Vuorinen, Joonas Kiili, Essi Alanko, Heini Huhtala, Jyrki Ollikainen, Piritta Setälä, Sanna Hoppu

**Affiliations:** 1 Faculty of Medicine and Health Technology, Tampere University, Tampere, Finland; 2 Department of Emergency, Emergency Medical Services, Centre for Prehospital Emergency Care, Anaesthesia and Pain Medicine, Tampere University Hospital, PO Box 2000, FI-33521 Tampere, Finland; 3 Faculty of Social Sciences, Tampere University, Tampere, Finland; 4 Department of Neurosciences and Rehabilitation, Tampere University Hospital, Tampere, Finland

**Keywords:** Emergency medical dispatch, Emergency medical services, Large vessel occlusion stroke

## Abstract

**Background:**

Emergency medical dispatchers typically use the dispatch code for suspected stroke when the caller brings up one or more symptoms from the face-arm-speech triad. Paramedics and emergency department physicians are trained to suspect large vessel occlusion stroke when the stroke patient presents with hemiparesis and cortical symptoms: neglect, aphasia, and conjugate eye deviation (CED). We hypothesized that these symptoms could be evident in the emergency call.

In this study, we aimed to describe common symptoms mentioned in the emergency calls for paramedic-suspected thrombectomy candidates. Secondly, we wanted to explore how the question about CED arises in the Finnish suspected stroke dispatch protocol. Our third aim was to find out if the symptoms brought up in suspected stroke and non-stroke dispatches differed from each other.

**Methods:**

This was a retrospective study with a descriptive analysis of emergency calls for patients with paramedic-suspected large vessel occlusion stroke. We listened to the emergency calls for 157 patients transported to Tampere University Hospital, a Finnish comprehensive stroke centre. Two researchers listened for symptoms brought up in these calls and filled out a pre-planned case report form.

**Results:**

Speech disturbance was the most common symptom brought up in 125 (80%) calls. This was typically described as an inability to speak any words (*n* = 65, 52% of calls with speech disturbance). Other common symptoms were falling down (*n* = 63, 40%) and facial asymmetry (*n* = 41, 26%). Suspicion of stroke was mentioned by 44 (28%) callers. When the caller mentioned unconsciousness the emergency dispatcher tended to use a non-stroke dispatch code. The dispatchers adhered poorly to the protocol and asked about CED in only 57% of suspected stroke dispatches. We found CED in 12 emergency calls and ten of these patients were diagnosed with large vessel occlusion.

**Conclusion:**

In cases where paramedics suspected large vessel occlusion stroke, typical stroke symptoms were described during the emergency call. Speech disturbance was typically described as inability to say anything. It is possible to further develop suspected stroke dispatch protocols to recognize thrombectomy candidates from ischemic cortical signs such as global aphasia and CED**.**

**Supplementary Information:**

The online version contains supplementary material available at 10.1186/s12873-022-00706-5.

## Background

Ischemic stroke causes an enormous burden when disability, mortality and financial aspects are considered [1]. A great deal of this burden is caused by large vessel occlusion (LVO) stroke [2]. The middle cerebral artery (MCA) nourishes most of the brain volume and surface area. Occlusion of its first segment, the M1-branch, is the most common target of endovascular treatment [3].

International treatment guidelines recommend that emergency medical services (EMS) develop methods to suspect LVO in patients with severe stroke symptoms [4, 5]. For this purpose, prehospital LVO scales have been formulated, but few have been tested in real life [6]. These LVO scales rely on the existence of cortical symptoms: conjugate eye deviation (CED), spatial neglect, and aphasia occurring alongside motor hemiparesis [7]. Among these cortical symptoms, CED has been shown to associate with large infarcts [8]. An exception to other prehospital LVO scales is the Stockholm Stroke Triage System in which the suspicion of LVO is solely based on the severity of motor hemiparesis [9]. In very recently published results from the RACECAT trial [10], Pérez de la Ossa et al. showed that the time from onset to groin puncture in patients with LVO significantly decreased when the EMS bypassed the primary stroke centre (PSC).

Timely recognition of stroke in the prehospital setting will accelerate time-critical interventions at in-hospital care [11–13]. The clinical management of stroke and accuracy of the dispatch criteria are among the most urgent research topics in prehospital care [14, 15]. CED and neglect were first included in the validation of a suspected stroke dispatch protocol by Krebes et al. [16]. Regrettably, the authors indexed patients only as having ischemic strokes or transient ischemic attacks and did not provide the number of LVOs. The PLUMBER Study [17] reported the prevalence of LVO in a population of dispatcher- and paramedic-suspected strokes. No publications describe the content of emergency calls of LVO patients, nor are there trials aiming to improve LVO recognition during the emergency call.

Our hypothesis was that aphasia and CED, at least, could be evident during the emergency call and if probed by the dispatcher would facilitate early detection of LVO. This could drastically change the EMS strategy by making it possible to activate a mobile stroke unit [18] or a helicopter [19] early in the triage and transport an LVO stroke patient directly to the comprehensive stroke centre (CSC). Unfortunately, in our previous publication [20], we concluded that the answer the dispatchers marked in the dispatch database to the CED question: ‘is the face or the gaze of the patient away from the side of the hemiparesis?’ did not recognize patients with LVO.

In this study, we describe common symptoms mentioned in emergency calls concerning patients with a paramedic-suspected LVO and primarily transported to our university hospital serving as a comprehensive stroke centre for one million people. In addition, we aim to determine why the dispatcher’s detailed question about CED is ineffective in recognizing LVO. Since the dispatcher asks the CED question only in suspected stroke dispatches, our third aim was to find out if the symptoms brought up in suspected stroke and non-stroke dispatches differed from each other.

## Methods

### Study design

This is a retrospective and descriptive analysis of emergency calls for patients with paramedic-suspected LVO.

### Setting

#### University hospital and emergency medical services

**Fig. 1:**
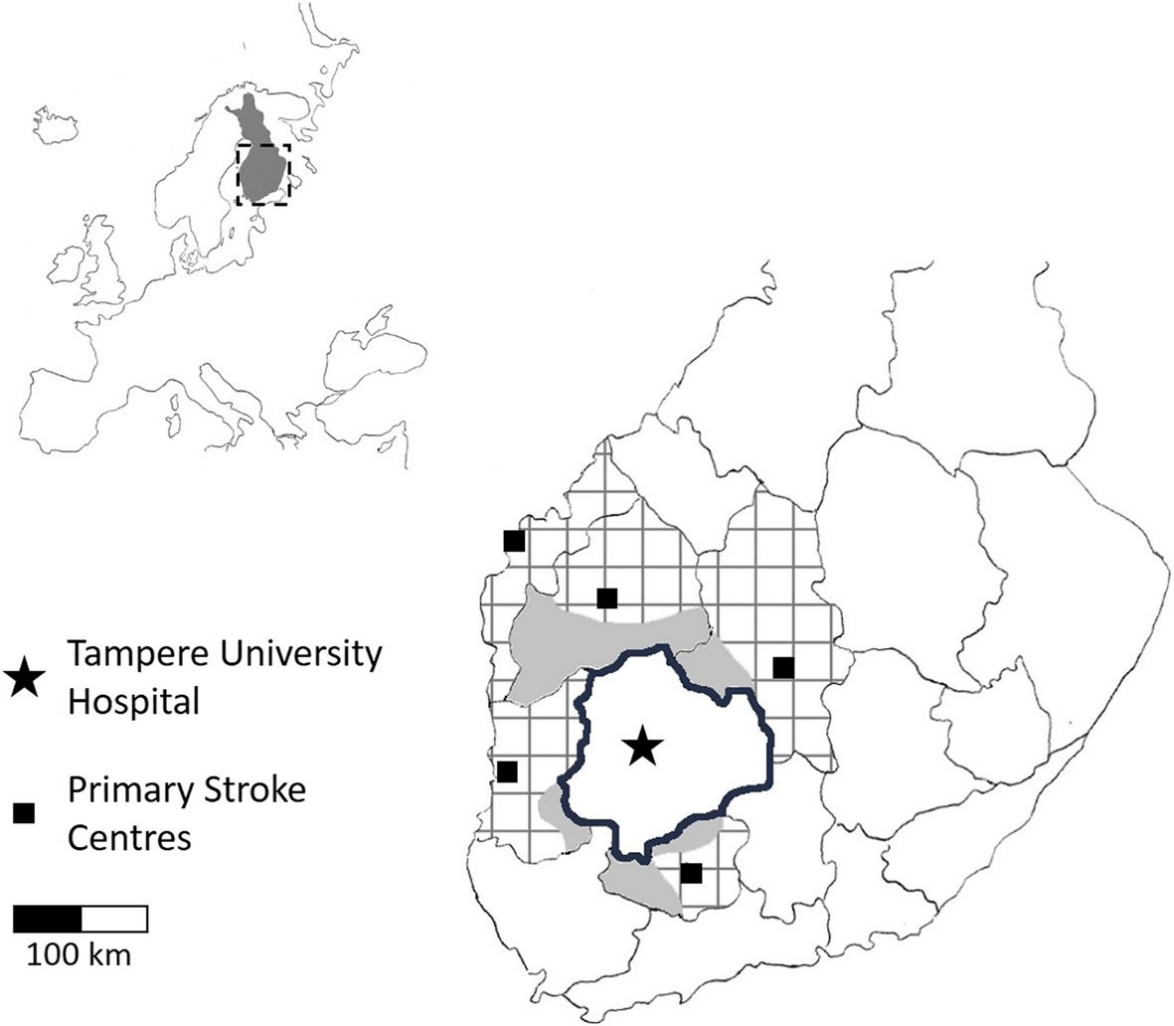
Map of the study area with a strengthened black line encircling Pirkanmaa hospital district, a square pattern indicating the hospital districts with primary stroke centres referring patients to Tampere University Hospital and solid grey areas representing the municipalities from which patients with suspected large vessel occlusion are transported straight to Tampere University Hospital

The emergency department of Tampere University Hospital serves as a CSC for a population totalling one million. The EMS transports all suspected stroke patients primarily to Tampere University Hospital from the Pirkanmaa Hospital District (Fig. 1). The EMS personnel from the imminent frontier of neighbouring hospital districts are trained to screen possible LVO strokes using the Finnish Prehospital Stroke Scale (FPSS) [21]. It is an easily memorized and binary categorized five-item screen for stroke patients. The first four items look for deficits in face, arm, leg and visual field. An abnormal finding in any of these items makes the patient a candidate for intravascular thrombolysis. The fifth item in FPSS is partial or forced CED in the opposite direction from any limb weakness. Paramedics consider a patient presenting with CED and any of the first four items as a candidate for mechanical thrombectomy. They consult the 24/7 on-call neurologist at the CSC to determine whether the patient should be diverted from the PSC and transported straight to the CSC, i.e., the mothership strategy [22]. Otherwise, patients from the five closest PSCs are transported to the CSC only after their eligibility for mechanical thrombectomy has been confirmed and intravenous thrombolysis potentially started in PSC, i.e., the drip-and-ship strategy [22]. A special thrombectomy alert is generated after the paramedic’s telephone consultation with the neurologist at the CSC. This alert is relayed to the personnel of the emergency department, those performing the computed tomography of the head, the neuro-interventional radiologist, the angio suite, the anaesthesia team and the high-dependency stroke unit. Our standard operating procedure states that primary targets for mechanical thrombectomy are the internal carotid artery, first and second branches of the middle cerebral artery, first branches of anterior and posterior cerebral arteries, and basilar artery. When anatomical conditions are suitable, also more peripheral parts of intracranial arteries may be considered. There were 178 mechanical thrombectomies done at Tampere University Hospital in 2020.

#### Emergency medical communication centre

All emergency calls in mainland Finland are operated by the Emergency Response Centre Agency Finland, which handles three million emergency calls per year. There are 445 dispatchers taking emergency calls in Finland. During 2018–19, the emergency call process was renewed. The computer-based Emergency Response Integrated Common Authorities (ERICA) was introduced to unite the dispatch system of all authorities: police, EMS, rescue services, emergency social welfare services and border guard. At the same time, the handling process of suspected strokes was revised. It resembles closely the Medical Priority Dispatch System card #28 [23]. According to the protocol, a suspected stroke is now dispatched to the EMS if the caller brings up the idea of a stroke or if one or more symptoms of the face-arm-speech triad are mentioned. At the time of data acquisition, the EMS responded immediately to suspected stroke dispatches with lights and sirens if the time from the last known point at which the individual was well was less than 6 h or if the patient woke up with stroke symptoms. Finally, and as a deviation from the card #28, ERICA guides the dispatcher to screen for a possible LVO by asking if the patient’s head or gaze tends to turn away from the side of the hemiparesis. The dispatcher has three options to mark as the answer to this question in the ERICA report: yes, no and unknown. It is not mandatory to ask the question, meaning the dispatcher may leave it unanswered. A positive answer to this question generates a separate notification to the EMS field commander. After the stroke dispatch, the emergency dispatcher tells the caller to make sure that the patient is at rest and that the scene is accessible.

### Study population

The study population consisted of emergency calls for patients with paramedic-verified CED and their prehospital prenotification generating a thrombectomy alert at the receiving hospital.

### Data collection

We went through the electronic patient records of all consecutive thrombectomy alerts at Tampere University Hospital emergency department after the deployment of ERICA (13th February 2019) until 31st August 2020 and reviewed the neurologist’s notes, the emergency department diagnoses and the radiologist’s report of the computed tomography angiography. We included EMS callouts coupled with these thrombectomy alerts where we could verify from the patient records that the paramedics confirmed CED during the EMS callout. We excluded patients who came from a PSC with a confirmed LVO and patients admitted to the emergency department from the wards of our own institution. The emergency call recordings and ERICA reports from these EMS callouts were requested from the Emergency Response Centre Agency Finland based on the date and time of the emergency call and the dispatch code generated from the call. The recordings were listened to, and a pre-planned case report form (Supplementary file) was filled out independently by two of the authors (P.V. and E.A.). P.V. is a specialist in anaesthesia and intensive care medicine. He has a subspecialty in prehospital medicine. E.A. is a second-year medical student. She has no previous contact with emergency medical services or the emergency response system. These results were then compared, and in case of disagreement, P.S. and S.H. were consulted to come to a consensus.

### Statistics

Categorical and continuous variables were analysed, and the results were used to identify differences between the suspected stroke and non-stroke dispatch groups. Categorical variables were analysed using cross-tabulation, and the χ^2^ test or Fisher’s exact test were carried out as appropriate. Continuous variables were analysed using the Mann-Whitney U-test. For the most common symptoms, 95% confidence intervals were calculated. Hypothesis testing was two-sided, and a *p*-value of ≤0.05 was considered statistically significant. Binary logistic regression modelling with forward stepwise selection (probability for entry ≤0.05, probability for removal ≥0.10) was used to aim to find out what symptoms are commonly brought up in the suspected stroke dispatches. IBM SPSS Statistics, version 27 (IBM Corp., Armonk, NY, USA) and Stata 16.0 (StataCorp, College Station, TX, USA) were used for statistical analyses.

### Ethics

The Ethics Committee of Tampere University Hospital supported the study design (ETL R20082R), and approval to view the patient records was granted by the hospital’s research director. The Emergency Response Centre Agency Finland granted a separate authorisation to access the emergency call recordings (HAK-21142). No interventions were performed and none of the patients were contacted in the study. Hence, the Ethics Committee of Tampere University Hospital waived the need for informed consent of the patients.

## Results

### Patients

**Fig. 2:**
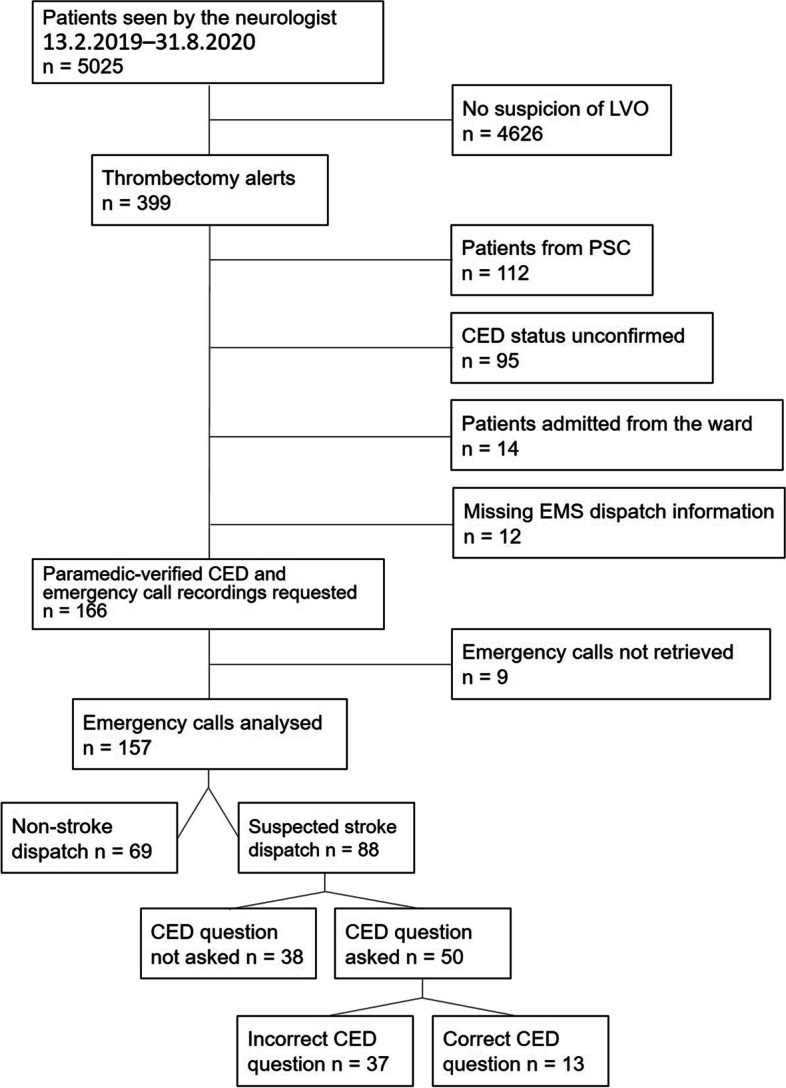
Patient flow. EMS, emergency medical services; CED, conjugate eye deviation; LVO, large vessel occlusion; PSC, primary stroke centre

During the study period, the neurologists working in the emergency department treated 5025 patients altogether, and among these patients, there were 399 patients with a thrombectomy alert. Of these cases, 157 had both a paramedic-verified CED and connected emergency calls. The patient flow is presented in Fig. 2.

Table 1 contains information about the patient demographics, dispatch details and emergency department diagnoses. The median age of the patients was 76 years (Q_1_–Q_3_: 69.5–83.1 years) and 85 (54%) of them were male. Most patients (*n* = 134, 85%) had a pre-existing condition diagnosed before the incident leading to the emergency call. The most common diagnosis made at the emergency department was acute ischemic stroke (*n* = 116, 74%), of which 95 had LVO (61% of all patients, 82% of ischemic strokes). Mechanical thrombectomy was performed on 79 patients (50% of all patients, 68% of ischemic strokes).

**Table 1:** Patient and emergency call characteristics

	Suspected stroke dispatch *n* = 88		Non-stroke dispatch *n* = 69		
	n/median	% /(Q_1_–Q_3_)	n/median	% /(Q_1_–Q_3_)	*P*-value
Male	47	53.4	37	53.6	0.979
Age, years	74.3	(67.2–82.7)	78.8	(70.7–83.7)	0.135
Medical history					
previously healthy	18	20.5	5	7.2	0.020
hypertension	57	64.8	53	76.8	0.102
atrial fibrillation	29	33.0	31	44.9	0.125
anticoagulation	24	27.3	18	26.1	0.868
diabetes	20	22.7	15	21.7	0.883
coronary disease	15	17.0	14	20.3	0.603
dementia	6	8.0	10	14.4	0.115
Caller					0.061
spouse	38	43.2	19	27.5	
outsider	24	27.3	21	30.4	
close relative	17	19.3	15	21.7	
healthcare professional	7	8.0	14	20.3	
indefinite	2	2.3	0		
Destination of EMS callout					0.063
private residence	68	77.3	46	66.7	
public place	15	17.0	11	15.9	
healthcare facility	5	5.7	12	17.4	
Diagnosis					0.009
LVO stroke	53	60.2	42	60.9	
non-LVO stroke	15	17.0	6	8.7	
intracerebral haemorrhage	20	22.7	14	20.3	
seizure	0		7	10.1	

*EMS* Emergency medical services, *LVO* Large vessel occlusion

### Dispatch information

The dispatchers suspected stroke in 88 (56%) of the emergency calls. Of non-stroke dispatches (*n* = 69, 44%), the most common codes were unconscious (*n* = 20, 29%), fall (*n* = 15, 22%), and cardiac arrest (*n* = 7, 10%). Regardless of the EMS callout dispatch code, the dispatch urgency was an immediate response with lights and sirens in 140 (90%) cases.

The onset time of symptoms was unknown to the caller in 71 (45%) calls. In those cases, the dispatcher decided to use a non-stroke dispatch code (*n* = 40) more often than a suspected stroke dispatch code (*n* = 31; *p* = 0.004).

### Symptoms described in the emergency calls

**Fig. 3:**
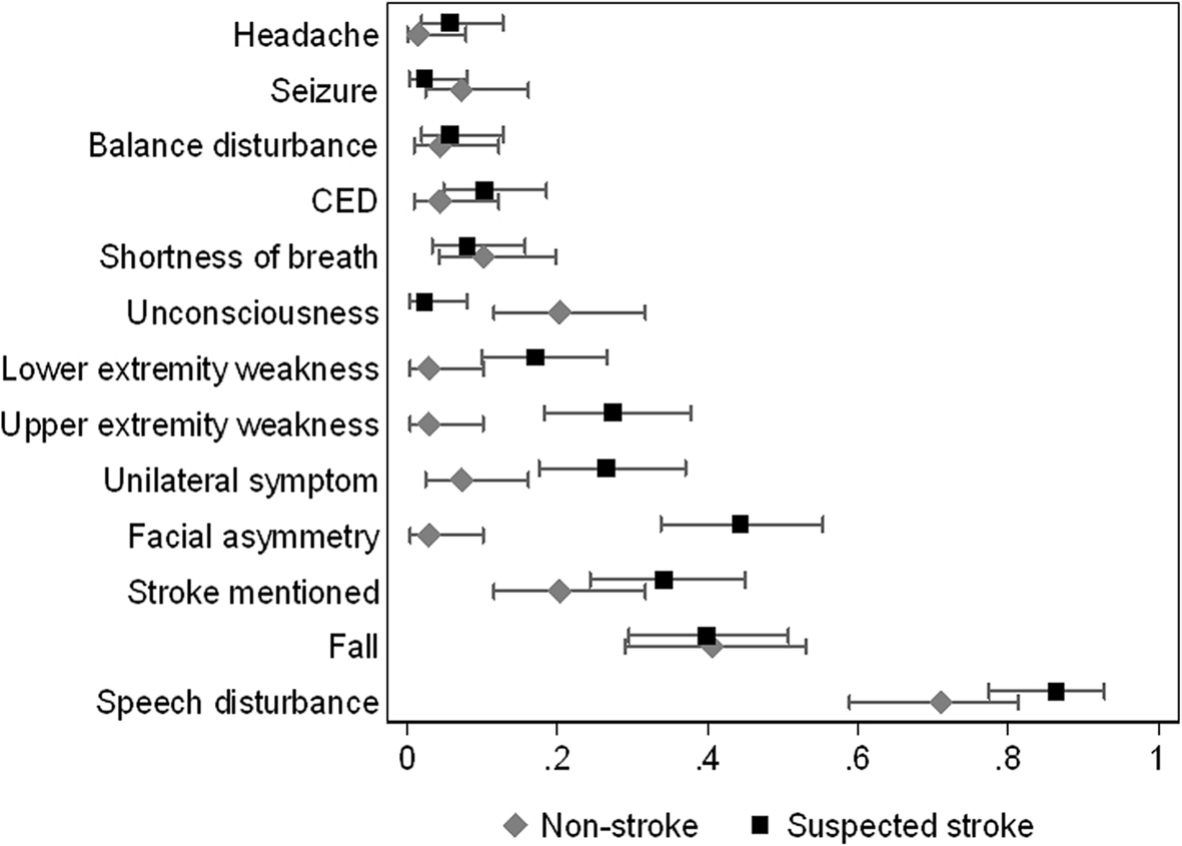
Proportion (with 95% confidence interval) most common symptoms mentioned in the emergency calls

In 44 (28%) emergency calls, the caller mentioned suspecting a stroke as the cause of the bout of illness. The most common symptoms during the emergency calls are presented in Fig. 3. In logistic regression modelling, the dispatcher preferred the dispatch code for suspected stroke over a non-stroke dispatch code when facial asymmetry (OR 30.0, 95% CI 4.9–185.0), upper (OR 13.7, 95% CI 2.3–81.6) or lower (OR 7.1, 95% CI 1.3–39.5) extremity weakness, or speech disturbances (OR 4.7, 95% CI 1.5–14.8) were mentioned. When unconsciousness came up in the emergency call, the dispatcher tended not to use the suspected stroke dispatch code (OR 0.05, 95% CI 0.006–0.47).

### Speech disturbance

Speech disturbance was by far the most common identifying symptom described in the emergency calls analysed. It was either spontaneously mentioned or inquired after by the dispatcher in 125 (80%) calls. Inability to form any words was mentioned 65 (41%) times in total. This was the case in 19 of the 20 (95%) cases when the dispatcher chose the dispatch code for “unconscious person”. The OR for using the dispatch code for “unconscious person” instead of suspected stroke code when the caller described that the patient is unable to speak anything was 38 (95% CI 4.9–290). Other examples of the description of speech disturbance were “unclear speech”, “incomprehensive” and “slurring”.

### Motor symptoms

The callers typically described motor symptoms with phrases like “the face of the patient is asymmetric”, “the other side is not working”, “the patient has lost strength in the right/left arm” and “the patient is unable to stand up”. In eight (5%) emergency calls, the dispatcher succeeded in guiding the caller to test the strength of the patient’s upper extremities and discovered the side of the hemiparesis correctly over the phone.

### Conjugate eye deviation

CED was asked about in 50 emergency calls (57% of the *n* = 88 suspected stroke dispatches), and the question was directed correctly 13 times, i.e., inquiring after the direction of the forced gaze. The callers mentioned CED in 12 (8%) of the *n* = 157 emergency calls analysed. In these 12 cases the dispatcher twice marked “no” in the ERICA report and twice left the question unanswered. Of the 12 patients with a positive CED answer, 11 were diagnosed with an ischemic stroke and of these 10 had LVO in computed tomography angiography.

Typical phrases in incorrectly placed CED questions were “is the patient forced to look in either direction?”, “is the patient able to look at you?” or “do see anything peculiar in the gaze of the patient?”

## Discussion

Our findings indicate that callers describe paramedic-suspected thrombectomy candidates as having typical stroke-associated symptoms, but dispatchers also use dispatch codes like “an unconscious person”, “a fallen person” or “cardiac arrest”. Among stroke symptoms, severity of speech disturbance, described as an inability to speak any words, could be a reasonable marker for LVO in emergency calls, but the dispatchers need further education since these patients were easily miscoded as being unconscious. In addition, caller-verified CED is valuable information that should be relayed to the EMS.

The emergency dispatcher’s sensitivity to recognize stroke ranges from 35% [24] to 80% and over [25, 26]. In Finland, Mattila et al. [27] recorded a sensitivity of 72% before ERICA was implemented. Yet, the level of the emergency dispatcher’s sensitivity is connected with the false positive rate, i.e., when one increases, so does the other. The positive predictive value of stroke in suspected stroke dispatches can fall as low as 20% [26]. In our study, the dispatchers had a 59% sensitivity in recognizing and using correctly the suspected stroke dispatch code for patients later diagnosed with ischemic stroke in the ED. Often, we found that inability to speak any words instead directed the dispatcher towards the “unconscious person” code. Interestingly, Mattila et al. [27] mention the caller describing speech disturbance as “He is not talking at all” in non-stroke dispatches but they fail to report the percentage of emergency calls with this feature. They also found that a fall at the onset of stroke was a predictor of the dispatcher using a non-stroke dispatch code. This is not consistent with our study. We found that the dispatcher used the code for suspected stroke, even though the caller mentioned the patient had fallen down. We have to keep in mind that these study samples are different. Mattila et al. [27] studied emergency calls for patients with diagnosed stroke or transient ischemic attack but we studied emergency calls for patients with paramedic-suspected LVO.

In almost all cases in our study, the dispatcher recognized the need for a high urgency dispatch. This was more notable than in an earlier study concerning Finnish stroke dispatch [27], possibly because the patients presented with more severe symptoms than in earlier studies. High urgency and immediate response are crucial factors in the successful treatment and management of stroke patients, though the dispatch code that is used is not inconsequential either. Paramedics act faster on the scene and adhere to neurological protocols better when dispatched with a suspected stroke code [11, 12]. These lead to better prehospital stroke recognition which hastens the patient’s in-hospital care [13].

We found that there is the potential to identify CED during the emergency call, but that dispatchers’ adherence to the protocol was disappointing. In the calls we studied, the CED question was either not asked or was asked but in a way that failed to elucidate the direction of the possible forced gaze. In our previous study [20] we did not have access to the emergency call recordings. We concluded in that study that the ERICA reports show that in 17% of suspected stroke dispatches the CED question is unanswered. In this present study, we found that the true percentage of unasked CED questions was 43%.

Further to this, the fact that dispatchers mismarked a positive answer for CED in the ERICA report shows a lack of proper implementation of the protocol. We also must keep in mind that inquiring about CED is instructed only in cases of suspected stroke, but a little less than half (41%) of the patients diagnosed with ischemic stroke in the ED were dispatched with a non-stroke dispatch code, meaning that the CED question never came up in ERICA during these calls. Going forwards, it is essential to decrease this portion of false-negative dispatch codes in stroke patients. We discovered three features in the emergency calls which typically led the dispatcher to choose a non-stroke dispatch: patients found with stroke symptoms after an unknown time from the onset of stroke, and emergency calls in which the caller mentioned that the patient was unconscious or unable to speak at all. These have to be taken into account when planning further education for the dispatchers.

In this research, we were delighted to note that eight dispatchers prompted the caller to find out whether the patient has motor symptoms. In each case, they correctly figured out the hemiparetic side. Previously, Mazya et al. [9] introduced a prehospital LVO scale based on the severity of the motor hemiparesis, as estimated during a teleconsultation. In their study, an ambulance nurse consulted the stroke physician at the CSC when they met a patient with a positive face-arm-speech -test. The stroke physician then assessed the severity of the hemiparesis on a scale from 0 to 2 for both, ipsilateral arm and leg. They reported a positive predictive value of 41% for this method to recognize LVO in cases in which the stroke physician considered the hemiparesis to be severe. This suggests that with proper education and dispatch protocol modifications, it could be possible to reliably deduce the side of the hemiparesis during the emergency call and whether the gaze of the stroke patient is directed away from this side. This could improve the possibility to discover LVO patients earlier and transport them directly to CSC.

### Strengths and limitations

Our second aim of this study was to find out why the CED question did not recognize LVO patients in our previous study [20]. Therefore, we deliberately chose to listen to emergency calls in which we could easily demonstrate that the paramedics had recognized the patient as a thrombectomy candidate on the grounds of CED. As such, we acquired 157 emergency call recordings out of 399 thrombectomy alerts. There is a possibility that larger-scale sampling could have produced a more accurate analysis. Yet, this work should be considered as a pilot study encouraging emergency dispatch centres review their protocols to include questions around CED and global aphasia in suspected stroke dispatch protocols.

Further to this, we did not look for neglect in the emergency calls since the dispatcher did not ask about it and we thought it would be difficult to recognize from the spontaneous narration of the caller. Yet, Zhao et al. [28] developed an algorithm for paramedics to use to spot neglect with a shoulder tap test if the patient’s left arm is weak. There are no reasons why this could not be incorporated into the dispatcher’s suspected stroke algorithm. A further limitation of this study is that we present no comparison to other neurological emergencies. To overcome this limiting factor, we recommend that future studies compare emergency calls for LVO patients with those for patients with distal occlusion strokes.

Nonetheless, the strength of this study is that—excluding one emergency call—all the callers were native Finnish speakers, and the quality of the recording was considered good or acceptable in each case.

## Conclusion

This is the first study to describe emergency calls for paramedic-suspected LVOs. We have demonstrated that paramedic-suspected thrombectomy candidates present with typical stroke symptoms in emergency calls. Cortical symptoms, most commonly speech disturbance described as an inability to speak any words, and to a lesser extent CED, are also evident. This demonstrates the potential for developing the dispatch protocols for a suspected stroke to recognize LVO in an emergency call. Appropriate training and simple instructions included in the stroke dispatch protocol could improve dispatchers’ accuracy. The dispatcher could, for example, encourage the caller to test if a patient, who is unable to say anything, presents with motor hemiparesis and thereafter enquire about CED. LVO identification at a very early stage would revolutionize the stroke triage.

## Supplementary Information

**Additional file 1.**

## Data Availability

Deidentified data will be preserved for 10 years after publication. Proposals for data sharing should be directed to the corresponding author by email.

## Abbreviations

CED: Conjugate eye deviation
CSC: Comprehensive stroke centre
EMS: Emergency medical services
ERICA: Emergency Response Integrated Common Authorities
FPSS: Finnish Prehospital Stroke Scale
LVO: Large vessel occlusion
MCA: Middle cerebral artery
PSC: Primary stroke centre
