# Supplementary material for: Cortical symptoms described in emergency calls for patients with suspected large vessel occlusion: a descriptive analysis of 157 emergency calls
Source: BMC Emerg Med. 2022 Aug 13;22:146. doi: 10.1186/s12873-022-00706-5 (PMC9375237; doi:10.1186/s12873-022-00706-5)
Supplement: Supplementary file 1 — Additional file 1. [file 12873_2022_706_MOESM1_ESM.docx]

| **Item** | **Examples** | **Choice** |
| --- | --- | --- |
| Caller | dropdown menu | daughter  son  spouse  healthcare professional  outsider  undetermined |
| Location | dropdown menu | private residence  healthcare facility  public place |
| Stroke mentioned | “stroke”, “suspected stroke”, “cerebrovascular accident” | y / n |
| Any speech disturbance mentioned | “inability to speak”, “slurring”, “unclear speech”, “difficulty to speak” | y / n |
| Speech disturbance is described as inability to speak | “unable to speak at all”, “no words”, “completely speechless” | y / n |
| Any symptom affecting only one side | “the other side is not moving” | y / n |
| Motor hemiparesis of upper extremity | “arm is weak”, “arm is not moving” | y / n |
| Motor hemiparesis of lower extremity | “leg is weak”, “unable to walk” | y / n |
| Problem with balance | “feels dizzy”, “unable to stand” | y /n |
| Visual disturbance | “suddenly lost vision” | y / n |
| Facial asymmetry | “the other side of the face is drooping”, “there is something wrong with his / her face” | y / n |
| Drooling | “is unable to swallow” | y / n |
| Falling | “fell down”, “tripped” | y / n |
| Unconsciousness | “is unconscious”, “is not awake” | y / n |
| Headache | “complains headache” | y / n |
| Malaise | “vomits”, “is about to throw up” | y / n |
| Seizure | “ fit”, “cramp”, “seizure” | y / n |
| Angina | “chest pain” | y / n |
| Dysrhythmia | “palpitations”, “arrhythmia” | y / n |
| Breathing difficulty | “unable to breath properly”, “gasping for air” | y / n |
| Any question about CED | “Is there something peculiar about the gaze of the patient?” | y / n |
| Correctly asked CED question | direction of the forced gaze is mentioned  “Is the face or the gaze of the patient away from the paretic side” | y / n |
| Callers answer to the CED question |  | y / n / not asked / not answered |
| Is the caller calm |  | y / n |
| Quality of the emergency call recording | dropdown menu | good  acceptable  poor  very poor (no common language) |
|  |  |  |

APPENDIX 1.

Items the researchers soughed in the emergency call recordings

CED: conjugate eye deviation, n: no, y: yes
